# Toward Predictive Models of Biased Agonists of the Mu Opioid Receptor

**DOI:** 10.1021/acs.biochem.4c00885

**Published:** 2025-04-10

**Authors:** Fernando J. Tun-Rosado, Elier E. Abreu-Martínez, Axel Magdaleno-Rodriguez, Karina Martinez-Mayorga

**Affiliations:** 1 Instituto de Química, Unidad Mérida, Universidad Nacional Autónoma de México, Carretera Mérida-Tetiz Km. 4.5, Ucú, Yucatán 97357, México; 2 Departamento de Física Aplicada, Centro de Investigación y de Estudios Avanzados, Unidad Mérida, Mérida, Yucatán 97310, México

## Abstract

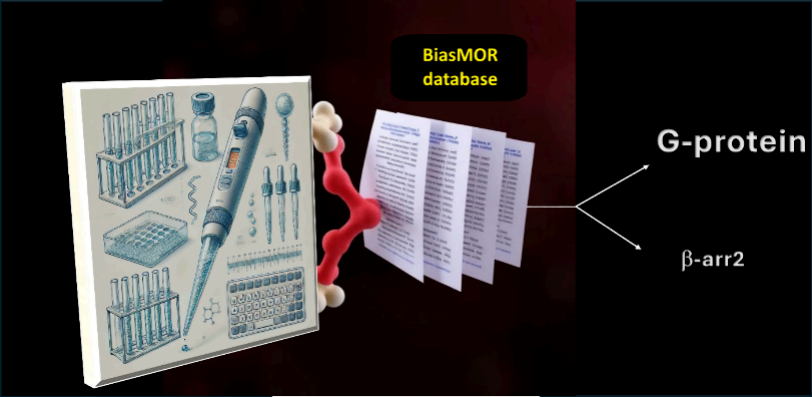

The mu-opioid receptor (MOR), a member of the G-protein-coupled receptor superfamily, is pivotal in pain modulation and analgesia. Biased agonism at MOR offers a promising avenue for developing safer opioid therapeutics by selectively engaging specific signaling pathways. This study presents a comprehensive analysis of biased agonists using a newly curated database, BiasMOR, comprising 166 unique molecules with annotated activity data for GTPγS, cAMP, and β-arrestin assays. Advanced structure–activity relationship (SAR) analyses, including network similarity graphs, maximum common substructures, and activity cliff identification, reveal critical molecular features underlying bias signaling. Modelability assessments indicate high suitability for predictive modeling, with RMODI indices exceeding 0.96 and SARI indices highlighting moderately continuous SAR landscapes for cAMP and β-arrestin assays. Interaction patterns for biased agonists are discussed, including key residues such as D^3.32^, Y^7.43^, and Y^3.33^. Comparative studies of enantiomer-specific interactions further underscore the role of ligand-induced conformational states in modulating signaling pathways. This work underscores the potential of combining computational and experimental approaches to advance the understanding of MOR-biased signaling, paving the way for safer opioid therapies. The database provided here will serve as a starting point for designing biased mu opioid receptor ligands and will be updated as new data become available. Increasing the repertoire of biased ligands and analyzing molecules collectively, as the database described here, contributes to pinpointing structural features responsible for biased agonism that can be associated with biological effects still under debate.

## Introduction

Opioid receptors (OR) belong to the superfamily of G-protein-coupled receptors (GPCRs). OR are essential for modulating pain and mediating analgesia. Among the four subtypes, the mu-opioid receptor (MOR) is the primary target of morphine and most opioid-based analgesics. Biased agonism at MOR, which refers to the selective activation of specific signaling pathways, has emerged as a critical area of research in the pursuit of safer opioid therapeutics.^1−4^ Predictive models of bioactivity are indispensable tools for discovering new molecules with optimized pharmacological properties. These models are widely utilized in both academic and industrial settings. However, their predictive accuracy hinges on multiple factors, particularly the quality and scope of the data used for model development.^5,6^ This makes the creation, maintenance, and documentation of molecular databases a foundational step in the modeling process.

Several publicly available molecular databases exist, with their selection often dictated by the specific goals of a study. Table 1 provides an overview of commonly used libraries in the GPCR field, detailing their content and data types. General libraries are versatile, supporting virtual screening and cheminformatics studies, while focused libraries cater to specific diseases or molecular types. A notable example is BiasDB,^7,8^ a manually curated database containing 727 cases of biased ligands. These ligands are categorized into four signaling pathways: G protein, G protein-selective, extracellular signal-regulated kinases (ERK), and β-arrestin. BiasDB is particularly relevant to this work, as it includes molecules that preferentially induce certain signaling effects, such as G-protein recruitment over β-arrestin, a phenomenon known as functional selectivity.

**Table 1:** Molecular Libraries Relevant in GPCR Research

database	description
BiasDB (https://biasdb.drug-design.de)	**BiasDB** is a manually curated database containing published biased GPCR ligands. Provides information about the chemical structure, target receptor, type of bias, assay categories used for bias determination, reference ligand, and literature source. Developed and maintained by the Dr. Gerhard Wolber lab at the Freie Universität Berlin, Institute of Pharmacy.
Helping to End Addiction Long-term (HEAL) Initiative Target and Compound Library (https://ncats.nih.gov/research/research-activities/heal/expertise/library)	**HEAL** Target and Compound Library contains compounds that are reported to modulate a variety of targets related to the perception of pain in the human body. This library was explicitly designed to omit controlled substances to prevent opioid-dominated screening results.
CheMBL (https://www.ebi.ac.uk/chembl/)	Bioactive drug-like small molecules (>2,000,000); includes 2D structures, calculated properties, and abstracted bioactivities (binding constats, pharmacology, and ADMET data). It is an easy-to-access database; searches can be defined by target (>11,000 targets) and browsed by activity type (EC_50_, *K*_i_, IC_50_, etc.).
GPCRdb (https://gpcrdb.org/)	The **GPCRdb** contains data, diagrams, and web tools for GPCRs. The user can browse all GPCR crystal structures and the largest collection of receptor mutants. Diagrams can be produced and downloaded to illustrate receptor residues (snake-plot and helix box diagrams) and relationships (phylogenetic trees). Reference (crystal) structure-based sequence alignments take into account helix bulges and constrictions, display statics of amino acid conservation, and have been assigned generic residue numbering for equivalent residues in different receptors.
IUPHAR/BPS Guide to PHARMACOLOGY (https://www.guidetopharmacology.org)	Provides quantitative information about drug targets, approved drugs, and experimental molecules of those targets. Depicts detailed data on targets. Created from a collaboration between The British Pharmacological Society (BPS) and the International Union of Basic and Clinical Pharmacology (IUPHAR).
GLASS GPCR (http://zhanglab.ccmb.med.umich.edu/GLASS)	**G**PCR-**L**igand **Ass**ociation (GLASS) is a manually curated repository for experimentally validated GPCR-ligand interactions. Retrieves information form literature and public database. Developed and maintained by the Zhang Lab at the University of Michigan, USA.

For the predictive modeling of biased agonism, it is essential that the database encompasses a wide range of activity, including both biased and unbiased (balanced) ligands. Before developing predictive models, a comprehensive qualitative analysis of structure–activity relationships (SAR) and exploratory data analysis is critical. In this study, we conducted a scaffold analysis, identified maximum common substructures, assessed modelability, and examined activity cliffs. Finally, we present an interactive pattern analysis of MOR-biased agonists as a strategy to guide the design of novel biased ligands.

## Materials and Methods

### Database Preparation

The initial set of ligands was obtained from the BiasDB database^7,8^ using the following criteria: receptor family (opioid receptor), receptor subtype (mu-opioid receptor), and bias category (G protein). Applying these criteria, BiasDB yielded a total of 68 compounds, all of which induce bias toward either G protein or β-arrestin signaling pathways. For these molecules, the following information was provided: name, SMILES representation, type of bias, and bibliographic references. However, information on potency (EC_5__0_) and maximum efficacy (E_max_) was not included. Consequently, a manual search was conducted to annotate the potency and efficacy values. Molecules were selected if they included relevant activity data against the MOR, such as affinity values (EC_5__0_, pEC_5__0_, or log EC_5__0_) or functional information.

The chemical structures were verified against the original references. Ambiguous and erroneous data were corrected, and missing data were added. Name and structural information in SMILES format were updated, and additional details—such as the type of assay, reference ligands used, and activity reported in experiments—were incorporated.

Regarding assay types, three primary assays for opioid receptors were considered: binding ([^35^S]GTPγS), cAMP accumulation, and β-arrestin recruitment. These are referred to as GTPγS, cAMP, and barr2 throughout this work. If the assay type was not explicitly mentioned, the original sources were consulted to confirm the information. Data on reference ligands used in the experiments were also added.

Activity values were reported as EC_5__0_, pEC_5__0_, log EC_5__0_, or pIC_5__0_ and documented as found in the original sources before unit standardization. Where possible, EC_5__0_ values were derived from pEC_5__0_ and log EC_5__0_ values. Molecules with activity reported in pIC_5__0_ were excluded due to the difficulty of conversion. Units were standardized to nanomolar (nM). A total of 36 molecules with undefined stereochemistry were recorded as racemic mixtures. This finalized database is referred to as BiasMOR in this work. BiasMOR is made available in the Supporting Information and contains the chemical structure in SMILES format and the activity values collected from the literature.

For comparative purposes, a data set of 201 molecules with binding affinity to MOR was downloaded from ChEMBL. This data set is referred to as BindingMOR. Additionally, a data set containing 10,885 approved drugs was downloaded from DrugBank for comparison.

### Structure–Activity Relationship (SAR) Analysis

The SAR analysis involved the generation of network similarity graphs (NSG) and the identification of the maximum common substructure (MCS) within clusters defined by Morgan fingerprints and a Tanimoto similarity cutoff of 0.9.

A systematic analysis of structure–activity relationships within the databases was performed by identifying the most frequently used scaffolds and comparing them with potency differences. Then, an NSG analysis was conducted.^9^ In NSG, compounds are represented as nodes connected by edges if their similarity exceeds the threshold (0.9). Nodes are color-coded and sized to indicate potency and activity, respectively. This approach helps identify structural features responsible for potency and highlights critical compounds or modifications.

The structure–activity relationship index (SARI)^10^ was calculated to quantify the continuity or discontinuity of the activity landscape. SARI categorizes SARs as continuous (low structural diversity with similar potency), discontinuous (activity cliffs, where structurally similar compounds show large potency differences), or heterogeneous. Using 2D structural similarities and potency data, continuity and discontinuity scores, normalized between 0 and 1, were calculated. High SARI values indicate continuous SARs, while low values denote discontinuous SARs. Intermediate scores suggest heterogeneous SARs. High continuity implies gradual activity changes across structurally diverse compounds. High discontinuity indicates sharp potency shifts among similar compounds.

The modelability of the databases was assessed using the RMODI index.^11,12^ which estimates the likelihood of achieving good predictive model performance based on neighbor and cluster dependencies and correlation coefficients. These metrics collectively provide insights into the suitability of the databases for developing predictive models.

## Results and Discussion

In this work, we developed and analyzed a curated database named BiasMOR, which includes MOR agonists measured for bias signaling, encompassing both biased and unbiased molecules. As a reference, we compared BiasMOR with another database, BindingMOR, which includes molecules with known MOR binding affinity. Following the structural and activity relationship analyses, we conclude this section by discussing interaction patterns of MOR-biased agonists developed over the years, comparing them to relevant literature models.

### Database Characterization and Distribution of Activity Values

**Figure 1:**
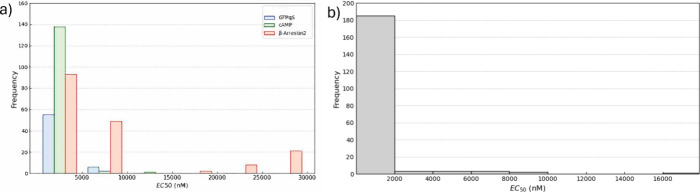
Distribution of activity values. (a) BiasMOR database. (b) Binding affinities for the BindingMOR database.

The distribution of activity values for BiasMOR is shown in Figure 1. Panel a displays the EC_50_ distributions for the GTPγS, cAMP, and β-arrestin (barr2) assays, each skewed to the left, with the barr2 assay showing slightly less skewness. For comparison, Panel b illustrates the distribution of EC_50_ values for BindingMOR, which is similarly skewed.

**Figure 2:**
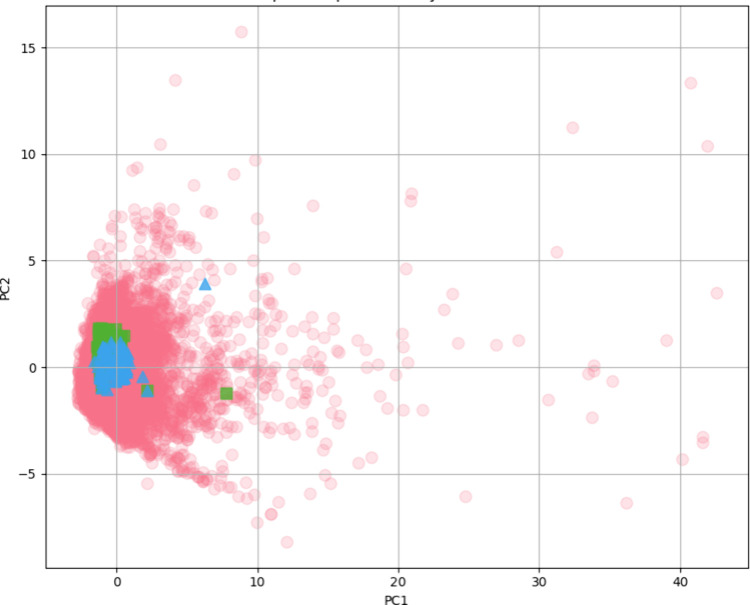
Two-dimensional representation of the chemical space of BiasMOR (green), BindingMOR(blue), and drug bank (red).

A broad comparison of physicochemical properties across BiasMOR, BindingMOR, and DrugBank-approved drugs is presented in Figure 2. The 2D chemical space visualization, based on Lipinski’s Rule of Five properties, reveals that biased ligands are concentrated within densely populated chemical space regions. Importantly, no significant physicochemical differences were observed between biased and opioid ligands, necessitating additional analyses to distinguish them.

### BiasMOR Database Curation and SAR Analysis

**Figure 3:**
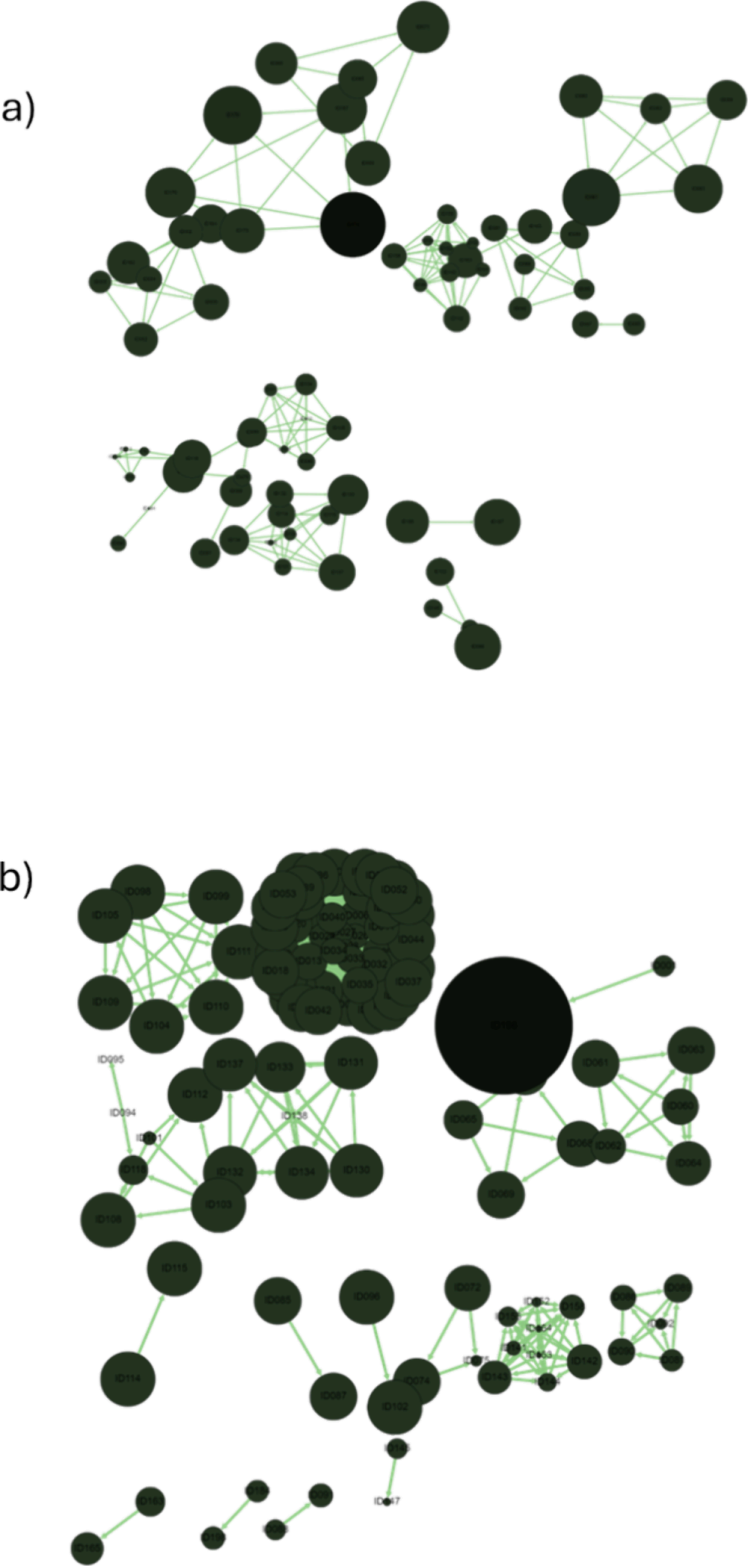
Network similarity graph of the BiasMOR database for the subsets (a) cAMP and (b) barr2. The smaller the node, the higher the potency.

BiasMOR initially contained 203 MOR agonists, reduced to 166 unique molecules after duplicate removal. Activity values were available for 62, 142, and 175 molecules in the GTPγS, cAMP, and barr2 assays, respectively. The network similarity graph (NSG) for the whole BiasMOR database compressed 123 nodes grouped into 20 clusters based on maximum common substructures (MCS), with 43 singletons. Figure 3 shows the NSG for the molecules with cAMP and barr2 activity values, panels A and B, respectively. Each node corresponds to one molecule. Similar molecules, based on their MCS, are connected with a line, and a group of connected nodes form a cluster. The smaller the node, the higher the potency. Thus, connected nodes with different sizes correspond to similar molecules with different activity values, known as activity cliffs.^13^ Clusters with even nodes are formed by similar molecules approximately equipotent; therefore, the activity landscape in that region is continuous. Panel a corresponds to molecules with measured cAMP value. It contains highly potent molecules (small nodes) but evenly distributed molecules with moderate activity. In turn, Panel b, barr2 data, contains small nodes, one large node, and the majority of the nodes are approximately evenly sized. Interestingly, the larger node is connected to a small node, which corresponds to an activity cliff. Also, notice that one cluster is highly populated with similar node sizes. This node shows a scaffold that has been heavily explored and has rendered molecules with similar potency.

The qualitative analysis described above can be complemented with modelability metrics shown in Table 2. The SARI values for the GTPγS, cAMP, and barr2 assays are 0.329, 0.577, and 0.534, respectively, suggesting a discontinuous activity landscape for GTPγS and moderately predictable SARs for cAMP and barr2 assays, coinciding with the qualitative analysis provided by the NSG. The RMODI values (>0.96 for all assays) indicate high modelability, making the data set well-suited for predictive model development.

**Table 2:** SARI and RMODI Values for the ChEMBL_opioids and biasMOR Datasets Using 1024-bit Morgan Fingerprints, Radius 2, and the Tanimoto Metric

		**BiasMOR**		
	**ChEMBL_opioids**	**GTPγS**	**cAMP**	**barr2**
**SARI**^a^	0.582	0.329	0.577	0.534
**RMODI**^b^	0.892	0.967	0.973	0.987

a Structure–activity relationship index.

b Regression modelability index.

The presence of activity cliffs largely depends on the end point under study. In the biasDB, there are three end points; however, not all compounds were evaluated in each of these, preventing a direct comparison of which assay is more prone to activity cliffs. Furthermore, it is unlikely that the same pair of molecules will constitute an activity cliff in more than one assay. Nonetheless, it is possible to identify activity cliffs in each end point and find coincidences. Exemplary cases are illustrated in Table 3. Note that subtle differences, such as stereochemistry and ring size, lead to activity cliffs in different assays. Since the design of biased agonists depends on both biological activities, the propensity of each assay to exhibit activity cliffs must be considered.

**Table 3:**
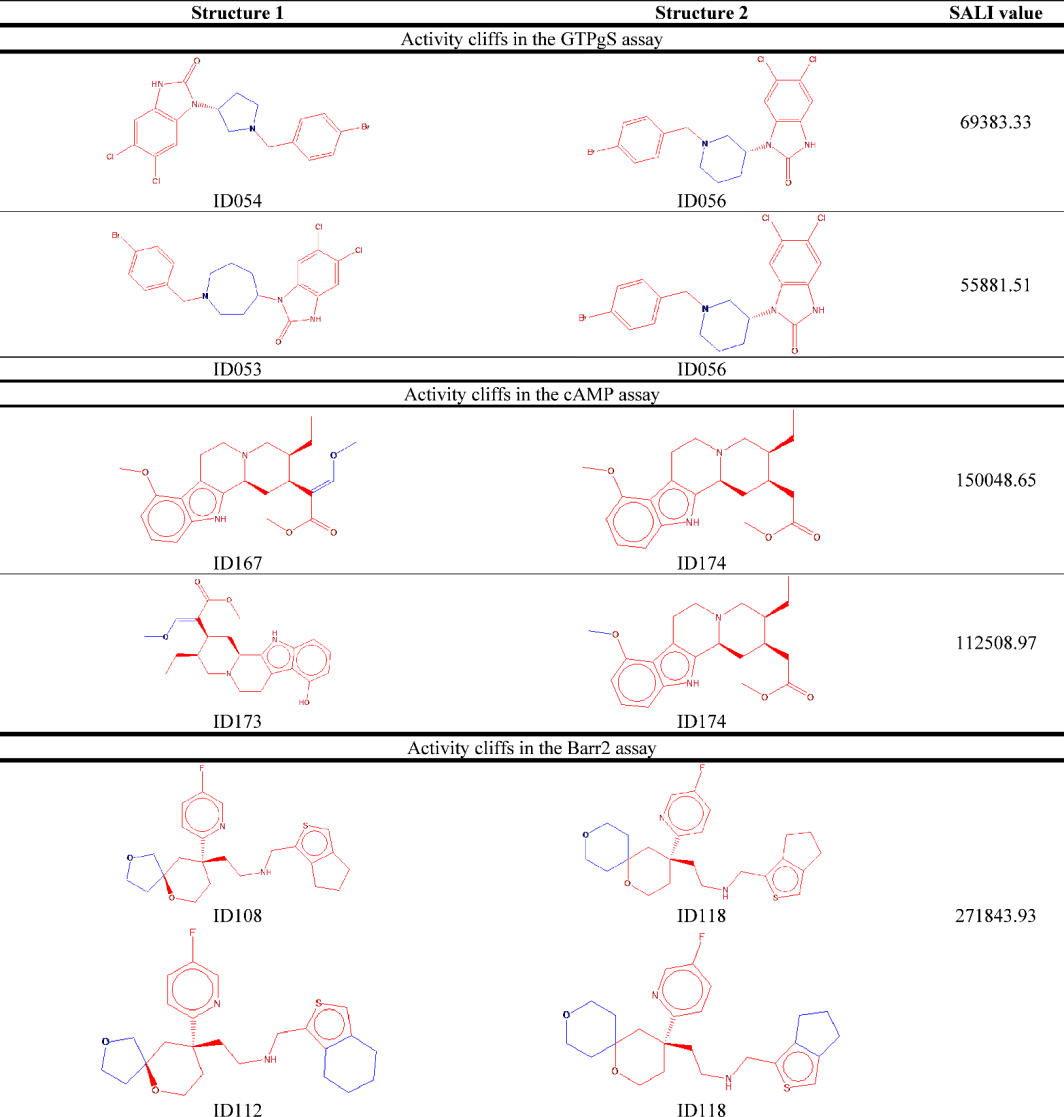
Exemplary Activity Cliffs Identified in the Different Assays, with Red and Blue Colors Indicating Commonalities and Differences in the Structures, Respectively

### BindingMOR Database Analysis

**Figure 4:**
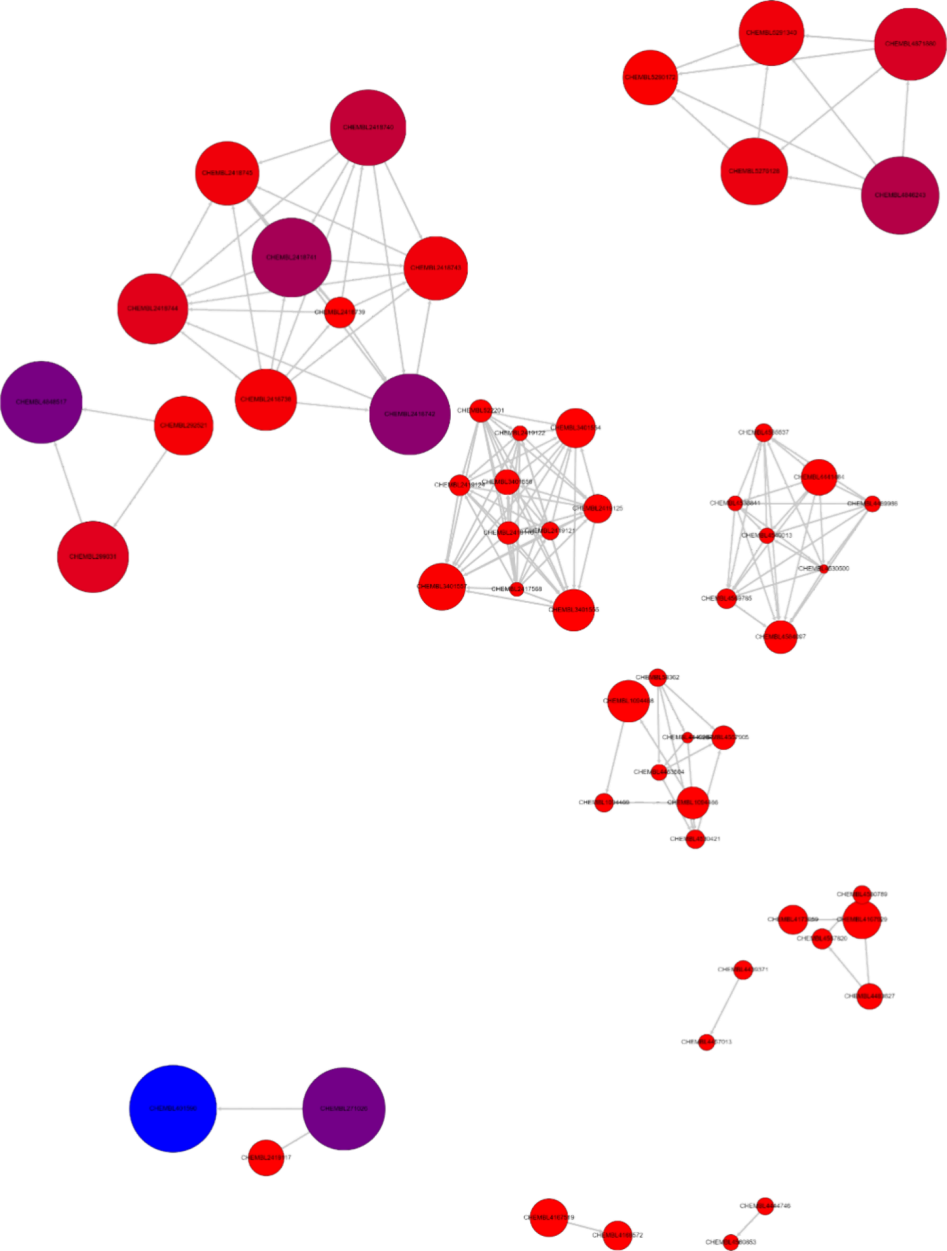
Network similarity graph of the BindingMOR database. The size and color of the nodes vary depending on the activity value of the compound. The most active compounds are represented by the smallest red nodes, while the less potent compounds are depicted as larger blue nodes.

The curated BindingMOR database contained 197 MOR ligands, reduced to 121 after removing compounds with incomplete activity data. The NSG for BindingMOR (Figure 4) comprises 58 nodes across 14 clusters, with 63 singletons. The SARI index (0.582) suggests moderately predictable SARs. Interestingly, some of the clusters with fewer nodes, contain active molecules, suggesting that further analysis of those scaffolds might provide active molecules in the binding affinity assay. Additionally, less active molecules (larger nodes) are grouped together, indicating a homogeneous inactive landscape for those scaffolds. Thus, a scaffold-based SAR analysis looks suitable. The RMODI (0.893) further supports the database’s suitability for robust predictive modeling.

Being bias signaling the result of the interaction between GPCRs and a particular group of agonists, analyzing interaction patterns is crucial. In the next section we summarize key interactions that are relevant for inducing bias agonism in MOR.

### Interaction Patterns for MOR-Biased Agonists

Building on our previous work on MOR-biased agonists,^14−16^ we characterized ligand–receptor interaction patterns using molecular docking, molecular dynamics (MD) simulations, and experimental validation. Key residues such as W318^7.35^ and W293^6.48^ were identified as critical for G protein signaling, while Y326^7.43^ contributed to both G protein and β-arrestin pathways. The allosteric sodium site (N150^5.35^ and D114^2.56^) was implicated in receptor regulation. Ligand-induced conformations were shown to vary based on ligand properties, stabilizing distinct receptor states.

Recent studies have validated these findings and expanded on enantiomer-specific interactions. For example, S-enantiomers preferentially interact with Y^7.43^ and D^3.32^, favoring β-arrestin signaling, while R-enantiomers engage differently, influencing G protein pathways. Comparative analysis highlights the significance of D^3.32^, Y^7.43^, and Y^3.33^ in biased signaling. This finding aligns with our previous computational models.

The SAR analysis indicated that binding affinity values could be explored based on scaffolds, and that biased ligands and enantiomers are prone to activity cliffs. These findings support the notion that biased agonism can be better understood through the analysis of binding recognition patterns. Thus, predictive models that incorporate interactive patterns are better prospects compared to those relying solely on ligand-based descriptors.

## Conclusions

This study integrates computational and experimental approaches to explore biased agonism at MOR. The curated BiasMOR database, with 166 unique agonists, provides a valuable resource for understanding structural and functional determinants of signaling bias. SAR analyses underscore the role of scaffold diversity and molecular features in modulating activity, with modelability indices confirming the database’s suitability for predictive modeling. Key residues such as D^3.32^, Y^7.43^, and Y^3.33^ are identified as critical for biased signaling, offering insights for designing next-generation opioid therapeutics. Collectively, these findings highlight the potential of integrated methodologies to advance the understanding of MOR biased agonism opening new avenues for the design of safer opioid drugs.

## Supporting Information Available

Chemical structures, in SMILES format, of 166 unique MOR ligands, annotated with GTPγS, cAMP, and barr2 activity values for 62, 142, and 175 molecules, respectively (XLSX)

## References

[ref1] Bohn L. M.; Lefkowitz R. J.; Gainetdinov R. R.; Peppel K.; Caron M. G.; Lin F.-T. Enhanced Morphine Analgesia in Mice Lacking β-Arrestin 2. Science 1999, 286 (5449), 2495–2498. 10.1126/science.286.5449.2495. 10617462

[ref2] Yang D.; Zhou Q.; Labroska V.; Qin S.; Darbalaei S.; Wu Y.; Yuliantie E.; Xie L.; Tao H.; Cheng J.; Liu Q.; Zhao S.; Shui W.; Jiang Y.; Wang M.-W. G protein-coupled receptors: structure- and function-based drug discovery. Signal Transduction Targeted Ther. 2021, 6 (1), 7 10.1038/s41392-020-00435-w. PMC7790836 33414387

[ref3] Zhang M.; Chen T.; Lu X.; Lan X.; Chen Z.; Lu S. G protein-coupled receptors (GPCRs): advances in structures, mechanisms and drug discovery. Signal Transduction Targeted Ther. 2024, 9 (1), 88 10.1038/s41392-024-01803-6. PMC11004190 38594257

[ref4] Cheng L.; Xia F.; Li Z.; Shen C.; Yang Z.; Hou H.; Sun S.; Feng Y.; Yong X.; Tian X.; Qin H.; Yan W.; Shao Z. Structure, function and drug discovery of GPCR signaling. Mol. Biomed. 2023, 4 (1), 46 10.1186/s43556-023-00156-w. 38047990 PMC10695916

[ref5] Martinez-Mayorga K.; Rosas-Jiménez J. G.; Gonzalez-Ponce K.; López-López E.; Neme A.; Medina-Franco J. L. The pursuit of accurate predictive models of the bioactivity of small molecules. Chem. Sci. 2024, 15 (6), 1938–1952. 10.1039/D3SC05534E. 38332817 PMC10848664

[ref6] Basith S.; Cui M.; Macalino S. J. Y.; Park J.; Clavio N. A. B.; Kang S.; Choi S. Exploring G Protein-Coupled Receptors (GPCRs) Ligand Space via Cheminformatics Approaches: Impact on Rational Drug Design. Front. Pharmacol. 2018, 9, 128 10.3389/fphar.2018.00128. 29593527 PMC5854945

[ref7] Bermudez M.; Nguyen T. N.; Omieczynski C.; Wolber G. Strategies for the discovery of biased GPCR ligands. Drug Discovery Today 2019, 24 (4), 1031–1037. 10.1016/j.drudis.2019.02.010. 30831262

[ref8] Omieczynski C.; Nguyen T. N.; Sribar D.; Deng L.; Stepanov D.; Schaller D.; Wolber G.; Bermudez M. BiasDB: A Comprehensive Database for Biased GPCR Ligands. BioRxiv 2019, 742643 10.1101/742643.

[ref9] Lounkine E.; Wawer M.; Wassermann A. M.; Bajorath J. SARANEA: A Freely Available Program To Mine Structure–Activity and Structure–Selectivity Relationship Information in Compound Data Sets. J. Chem. Inf. Model. 2010, 50 (1), 68–78. 10.1021/ci900416a. 20053000

[ref10] Peltason L.; Bajorath J. SAR Index: Quantifying the Nature of Structure–Activity Relationships. J. Med. Chem. 2007, 50 (23), 5571–5578. 10.1021/jm0705713. 17902636

[ref11] Luque Ruiz I.; Gómez-Nieto M. Á. Regression Modelability Index: A New Index for Prediction of the Modelability of Data Sets in the Development of QSAR Regression Models. J. Chem. Inf. Model. 2018, 58 (10), 2069–2084. 10.1021/acs.jcim.8b00313. 30205684

[ref12] Luque Ruiz I.; Gómez-Nieto M. Á. Study of Data Set Modelability: Modelability, Rivality, and Weighted Modelability Indexes. J. Chem. Inf. Model. 2018, 58 (9), 1798–1814. 10.1021/acs.jcim.8b00188. 30149700

[ref13] Maggiora G. M. On Outliers and Activity CliffsWhy QSAR Often Disappoints. J. Chem. Inf. Model. 2006, 46 (4), 1535 10.1021/ci060117s. 16859285

[ref14] Hernández-Alvarado R. B.; Madariaga-Mazón A.; Cosme-Vela F.; Marmolejo-Valencia A. F.; Nefzi A.; Martinez-Mayorga K. Encoding mu-opioid receptor biased agonism with interaction fingerprints. J. Comp. Aided Mol. Des. 2021, 35 (11), 1081–1093. 10.1007/s10822-021-00422-5. 34713377

[ref15] Marmolejo-Valencia A. F.; Martínez-Mayorga K. Allosteric modulation model of the mu opioid receptor by herkinorin, a potent not alkaloidal agonist. J. Comp. Aided Mol. Des. 2017, 31 (5), 467–482. 10.1007/s10822-017-0016-7. 28364251

[ref16] Marmolejo-Valencia A. F.; Madariaga-Mazón A.; Martinez-Mayorga K. Bias-inducing allosteric binding site in mu-opioid receptor signaling. SN Appl. Sci. 2021, 3 (5), 566 10.1007/s42452-021-04505-8.

